# Long-term outcomes of titration-guided focal selective retina therapy for chronic central serous chorioretinopathy: retrospective real-world data analysis

**DOI:** 10.1186/s12886-025-04581-z

**Published:** 2026-01-08

**Authors:** Migle Lindziute, Maximilian Binter, Nizar Al Mahamed, Amelie Pielen, Carsten Framme, Jan Tode, SRT study group, Marita Awe-Krüger, Maximilian Binter, Anke Beckmann, Jan Hendrik Brahms, Ralf Brinkmann, Reginald Birngruber, Pascal Buley, Maximilian Büttner, Lisa Danzmann, Nicolas Feltgen, Carsten Framme, Marten Gehlhaar, Thomas Gröber, Melanie Haar, Jonas Herden, Hans Hoerauf, Christina Sophie Jacobsen, Bernd Junker, Katja Kleemann, Katharina Knoll, Vanessa Kübler, Benjamin Luger, Yoko Miura, Wasim Abou Moulig, Migle Lindziute, Ulrike Peters, Amelie Pielen, Johann Roider, Christopher Rosenstein, Eric Seifert, Nina-Antonia Striebe, Dirk Theisen-Kunde, Jan Tode, Ingo Roland Volkmann

**Affiliations:** 1 Hannover Medical School, University Eye Hospital, Carl-Neuberg-Str. 1, 30625 Hannover, Germany; 2 Augennord, Ophthalmological Practice, Eckernfoerde, Germany; 3 Maximilians Augenklinik, Nürnberg, Germany

**Keywords:** Ophthalmology, Selective retina therapy, Titration-guided, Focal laser therapy, Chronic central serous chorioretinopathy, Re-treatment, Subretinal fluid, BDVA

## Abstract

**Objectives:**

This retrospective study aimed to evaluate the effect of titration-guided focal selective retina therapy (SRT) for chronic central serous chorioretinopathy (cCSCR) and determine the need and effectiveness of re-treatment.

**Methods:**

SRT was performed in 60 eyes of 57 patients with cCSCR, targeting focal leakage points (FLP) using a Nd:YLF-Laser at 527 nm (R:GEN®, Lutronic, South Korea) titration-guided at 80% threshold, with 140 ms irradiation time, 100 Hz frequency, 1.7 µs pulse duration, 200 µm spot size. Best documented visual acuity (BDVA), peak height of subretinal fluid (SRF), height of SRF at the fovea, central retinal thickness (cRT) and total macular retinal volume (tRV) were measured.

**Results:**

A significant improvement in BDVA was observed up to 1 year compared to baseline (*p* < 0.02). A limited analysis due to the small sample size showed no significant difference between the BDVA at baseline and 2-year (*n* = 15) and 3-year (*n* = 10) follow-ups. A significant decrease in the height of the SRF at the highest point and fovea and total retinal volume up to the 3-year follow-up was observed compared to the baseline (*p* < 0.03). Thirty (50%) eyes of 30 patients required re-treatment, on average 9.2 ± 9.6 months after the initial SRT. This study found no predictors for the need of re-treatment.

**Conclusions:**

The study concludes that SRT is safe and shows anatomical and functional benefits for treating cCSCR. We recommend performing regular check-ups every three months and following a zero-fluid tolerance policy for re-treatment.

**Trial registration:**

German Clinical Trials Register ID: DRKS00031038, registration date: 2023-01-16

## Background

Central serous chorioretinopathy (CSCR) is a common disease of the posterior segment of the eye with an incidence of 10 per 100,000 men and 2 per 100,000 women [1]. The average age is 40 to 50 years [2]. Typical presentation includes a dome-shaped neuroretinal detachment due to subretinal fluid (SRF), retinal pigment epithelium (RPE) dysfunction, and choroidal hyperpermeability and thickening. Patients suffer from central vision loss, scotoma, micropsia or metamorphopsia, hypermetropisation, and reduced contrast sensitivity [1].

There is no international consensus on classification of this disease. Most authors distinguish between an acute form, which is self-limiting within 4 months, and chronic central serous chorioretinopathy (cCSCR). However, this classification oversimplifies the disease’s complexity, such as the differences between diffuse or focal leakage [2]. Nevertheless, it is useful in guiding therapy as prolonged SRF lasting over 4 months can lead to irreversible visual loss and retinal atrophy [3]. Acute CSCR frequently resolves spontaneously and approximately 16% of all cases progress to the chronic stage [1, 4].

The precise pathogenesis of the disease remains unclear, and several theories are discussed. The general assumption is that the disease is caused by changes in the choroid that result in damage to the RPE and photoreceptors [2]. The exact site of primary choroidal changes is debated. One theory suggests that the main cause is ischemia in the choriocapillaris resulting in diffuse or focal damage in the RPE leading to SRF [1]. Another suggests that disturbances in choroidal autoregulation, leading to chronic venous stasis, and hyperpermeability of the larger choroidal vessels [5]. The greatest external risk factor for CSCR is use of corticosteroids [2]; both mineralocorticoid and glucocorticoid are considered important in the pathogenesis [1].

The complex clinical picture and the not conclusively clarified pathogenesis complicate the search for optimal therapy guidelines. Preventing long-term RPE damage by fully resolving SRF should be the primary goal of treatment [3]. However, due to the high spontaneous remission rate with good visual prognosis, therapy should most importantly feature a good safety profile.

Because of the suspected role of mineralocorticoids in CSCR, the VICI study tested the effect of eplerenone in a randomized, double-blind, placebo-controlled trial. It was shown that eplerenone was not superior to placebo in people with cCSCR after 12 months of treatment and should no longer be prescribed [6].

Conventional thermal laser photocoagulation should target only extrafoveal or even extramacular leakage points, as vision loss, scotoma, caused by the destruction of the neurosensory retina, reduced contrast sensitivity, and/or choroidal neovascularization can occur [2, 7]. Regenerative Retinal Laser and Light Therapies (RELITE), which are laser therapies that promote regeneration of the choroid/RPE/photoreceptors, that spare neural retina [8, 9]. They have hence been studied as treatment for CSCR.

RELITE includes photothermal, photochemical, photodisruptive laser therapies and photobiomodulation. The first three mentioned, have been applied as CSCR treatment.

Photothermal laser therapy (PTL) has mostly been applied by microsecond pulsed lasers. Unlike conventional lasers, the micropulse technique enables heat to dissipate between pulses and reduces unintended tissue damage. The temperature remains below the threshold for denaturing cellular proteins, avoiding laser burns [2]. A review of subthreshold micropulse laser for CSCR highlights its efficacy in improving both retinal morphology and visual function compared to no treatment or photodynamic therapy [10].

The second RELITE category and most commonly used therapy for CSCR is a photochemical approach, photodynamic therapy (PDT). This treatment involves an intravenous injection of verteporfin, followed by irradiation of the affected area of the retina. Free radicals are formed, particularly in the choriocapillaris, which lead to damage of the vascular endothelium and thus hypoperfusion [11]. As a result, the vessels in the capillary bed under the damaged RPE undergo remodeling [12]. This therapy has the advantage of high selectivity and spares the retinal photoreceptors. One drawback of PDT therapy is the risk of systemic photosensitivity [2]. The PLACE trial, a large multicenter, prospective randomized controlled trial compared half-dose PDT to micropulse laser treatment in cCSCR and found a higher rate of SRF resolution and better functional outcome after half-dose PDT [5]. However, atrophy of RPE and choroidal ischemia have been reported as side effects after PDT with verteporfin [13]. Therefore, long-term complications need to be studied in the future.

As third RELITE category, photodisruptive laser therapy, selective retina therapy (SRT) with pulse durations at about 1.7 µs results in RPE cell disruption by intracellular microbubble formation, sparing the neuroretina. The microvaporization-induced local RPE defects are closed by the proliferation and migration of neighboring RPE cells [14]. These remodeling processes may contribute to restructuring of the blood-retinal barrier and improve the metabolic function of RPE [15]. Regenerating RPE cells restore tissue integrity within 1–2 weeks post-irradiation [16]. No side effects of this procedure have been described so far [14, 15, 17, 18]. A limitation of SRT is the lack of defined optimal irradiation parameters, application patterns of laser foci and treatment strategy, currently based only on expert opinion and conventional photocoagulation. [19]. SRT has shown therapeutic success in CSCR and diabetic macular edema [7, 15, 20]. However, chronic cases of CSCR have been found to be less responsive to SRT treatment compared to acute cases. Nevertheless, promising outcomes have been observed with re-treatment in such cases [21].

The goal of this study was to evaluate the long-term effect of SRT in cCSCR as well as evaluate the need and effectiveness of re-treatment.

## Methods

### Subject selection

Patients with cCSCR, who showed visible leakage spots in fluorescein angiography (FA) were included in this retrospective study. This study defined cCSCR as ongoing symptoms for at least 4 months with persistent SRF, which was verified with spectral-domain optical coherence tomography (OCT). Each patient underwent a detailed ophthalmologic examination including best documented visual acuity (BDVA), slit-lamp examination, tonometry, indirect ophthalmoscopy, OCT of the macula, and FA. The study was conducted in accordance with the Declaration of Helsinki and approved by the Institutional Ethics Committee of Hannover Medical School (Ethics Approval Nr. 7393; 2017–04-06). Informed consent was obtained from all subjects involved in the study.

### SRT protocol

Treatment was performed using a slit-lamp-based selective retina therapy laser (R:GEN®, Lutronic, South Korea; CE-certified according to 93/42 MDD) that meets the requirements of the European Union Medical Device Regulation requirements 93/42 EEC. It is a 527 nm-wavelength frequency-doubled Nd: YLF laser. The procedure used an irradiation time of 140 ms at a frequency of 100 Hz, a pulse duration of 1.7 µs, and a spot size of 200 µm. Each SRT exposure was composed of 15 pulses in succession. Before beginning macular therapy, titration lesions with a starting energy of 50 µJ were administered adjacent to the arcades in ascending intensity, rising in 10 µJ increments until a noticeable whitening effect was seen funduscopically. The final treatment for the focal leakage points used energy at 80% of the threshold energy level. The titration was done to make sure there was enough energy to destroy the RPE without harming the photoreceptors. Energy was controlled utilizing the optoacoustic feedback in ramping mode throughout the delivery of each treatment spot. This feedback mechanism is integrated in the system. A contact lens sensor measures acoustic waves emitted by rupturing RPE cells, thereby reporting the desired therapeutic effect. Thus, the energy-level applied was monitored and then adjusted if feedback reported under- or overtreatment. The automatic termination mechanism for laser irradiation, controlled by real-time feedback dosimetry was not utilized.

A single SRT spot was placed at the center of the RPE leakage area, even if the fovea was involved, with additional laser spots arranged in a circular pattern around it until the entire leakage area was treated, except in cases with very small FLPs ( < 100 µm), where less spots were applied. Laser spots were manually applied with an intended spacing under 0.5 spot diameters. Because of subtle eye movements and no intraoperative visibility of SRT lesions, effective spacing varied between confluent and approximately half a spot diameter. Overlap was deliberately avoided. All treatments were performed by a small number of experienced specialists, which helped to reduce inter-operator variability.

Re-treatment was performed in cases where SRF showed no reduction or an increase compared to baseline, together with a visible FLP. Eyes with partial resolution of SRF were not re-treated at that time. Not all non-responder eyes underwent immediate re-treatment, as treatment decisions were also influenced by patient preference and cautious re-treatment policy at that time.

### Clinical examination and analysis

Patients were examined at baseline, 1, 3, 6, 12 months after SRT. Patients were offered voluntary follow-up examinations at the 2- and 3-year marks. The primary endpoints of this study were improvement of BDVA and SRF resolution. BDVA was examined at each visit and converted to logMAR for statistical analysis [22]. Fundus autofluorescence (FAF), FA, and OCT were performed using Heidelberg Engineering Spectralis OCT (Heidelberg Engineering, Heidelberg, Germany). In some cases, FA was not performed at each follow-up visit because patients had previously experienced mild adverse reactions and preferred to avoid repeat testing. In the case of re-treatment, it was noted whether the focal leakage point (FLP) was persistent, recurring, or new.

The distance from the fovea to the nearest FLP, peak height of SRF and height of SRF at the fovea were measured. Central retinal thickness (cRT) in central 1 mm of the ETDRS grid, and total macular retinal volume (tRV) of the complete ETDRS grid were measured in the retinal thickness map of dense volume macular SD-OCT scans. To further characterize treatment outcomes, eyes were stratified according to their subretinal fluid (SRF) response. Based on changes in SRF height at the highest point relative to baseline, three categories were defined: complete resolution, partial resolution (defined as > 25% reduction in SRF height), and non-responders (≤25% reduction in SRF height). The distribution of eyes across these response categories was assessed at each follow-up timepoint.

### Statistical analysis

Statistical analysis of data obtained in the study was performed using Microsoft Excel 2019 (Microsoft Corporation, Redmond, WA) and Statistical Package for Social Sciences (SPSS) version 27.0 (IBM Corp., Armonk, NY, USA). Graphs were created using GraphPad Prism Ver. 8.4.3 (GraphPad Software Inc., La Jolla, CA).

The one-sample binomial test was used to determine if the proportion of subjects in one of two categories was significantly different from 0.5. The Shapiro-Wilk test was used to analyze the normality of the distribution of empirical data. Student t-test was performed to test the equality of means in two and one-way ANOVA in more than two normally distributed populations. The Wilcoxon rank test was used to compare the means of matched samples in not normally distributed data. The Mann-Whitney U Test was used to compare the equality of data in two groups with not normally distributed data. Fisher’s exact test was used to analyze differences in two groups when the dependent variable was measured at a nominal level. The Z-test was used when comparing the difference in population proportions between two groups. The Spearman correlation coefficient was used to measure the linear correlation between variables. The Kaplan–Meier analysis was used to estimate the probability of an event, such as the need for re-treatment, over a certain period of time. Cox regression test was used to evaluate the effect of covariates on the probability of an event during a certain time period. Binary logistic regression analysis was used to evaluate the relationship between a set of predictor variables and a binary outcome variable. All means are presented with standard deviations (Mean±SD). Results with a significance level of *p* < 0.05 were interpreted as statistically significant.

## Results

### Demographic data

A total of 60 eyes (subjects) of 57 patients were included in the analysis. We did not include patients if the RPE leakage was outside of the vascular arcades. Three patients (2 male and 1 female) had cCSCR in both eyes at separate time intervals and received SRT treatment on each eye. 55 (92%) subjects were followed up to 3 months, 40 (67%) up to 6 months, 31 (52%) up to 1 year, 15 (25%) up to 2 years and 10 (17%) up to 3 years. Two patients skipped their 1-month follow-up visit.

There were 53 male and 7 female subjects included in the study. The study included statistically significantly more men with cCSCR (*p* < 0,001). The average age of subjects was 48.3 ± 11.2 years (minimum 30, maximum 76).

### SRT treatment characteristics

The average treatment energy was 146.2 ± 26.1 µJ (minimum 95, maximum 210). An average of 10.2 ± 6.1 (minimum 4, maximum 37) SRT lesions were applied on an average of 1.3 ± 0.6 (minimum 1, maximum 4) FLPs. A single FLP was irradiated with an average of 8.2 ± 3.9 laser spots (minimum 2, maximum 23). The nearest to the fovea FLP was on average 1408 ± 993 µm (minimum 220 µm, maximum 4336 µm) away from the center of the fovea at baseline.

### Course of the disease

Out of all subjects, 23 (38%) reported having symptoms like decreased visual acuity, scotoma, hypermetropisation and metamorphopsia for less than 6 months before diagnosis, 9 (15%) from 6 to 12 months, 7 (12%) from 1 to 2 years, and 21 (35%) for more than 2 years before diagnosis. Treatment with SRT was performed at less than 6 months after the beginning of symptoms in 10 (17%) of subjects, from 6 to 12 months in 14 (23%) of cases, from 1 to 2 years in 12 (20%) and more than 2 years before SRT in 24 (40%) of cases.

**Fig. 1:**
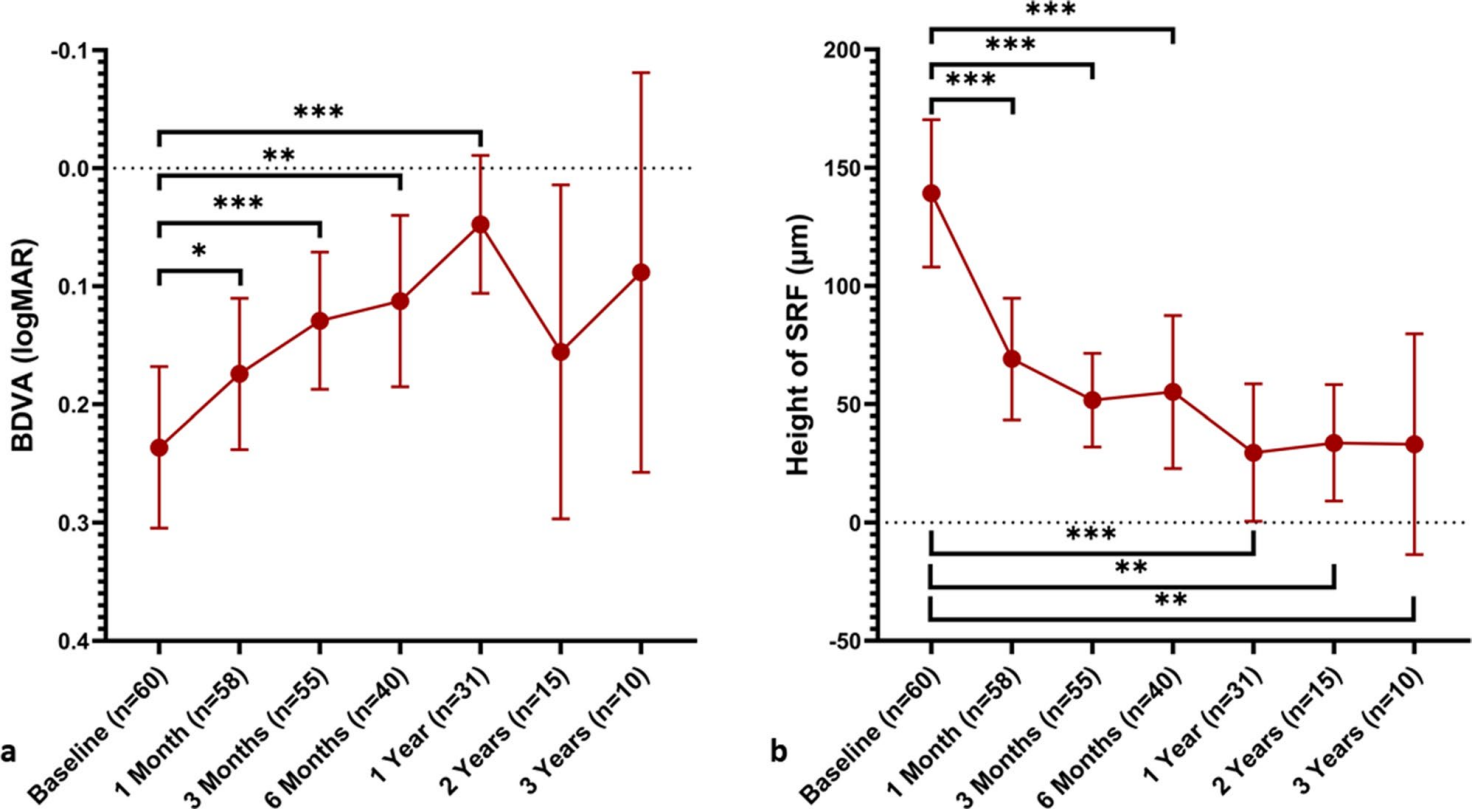
BDVA (logMAR) (**a**) and mean foveal SRT height (**b**) over a 3-year follow-up period. Mean values are plotted and the 95% confidence interval is displayed as error bars, n-values are presented below the x-axis indicate the number of eyes examined at each timepoint. Wilcoxon rank test was used to compare between matched samples. * *p* < 0.05, ** *p* < 0.01, *** *p* < 0.001

Subjects BDVA, height of SRF at the highest point and at the fovea, central retinal thickness as well as total retinal volume were measured at baseline and each follow-up examination (Table 1). Statistically significant improvement in BDVA was observed at each follow-up examination up to the 1-year follow-up when compared to baseline (*p* < 0.02). There was no significant difference when comparing BDVA at baseline to the 2-year and 3-year follow-up data in the smaller groups that attended these follow up examinations (Fig. 1). A significant decrease in the height of SRF at the highest point and at the fovea as well as a decrease in tRV was observed at all follow-up examinations up to the 3-year follow-up when compared to baseline (*p* < 0.03). Decrease in cRT was observed at follow-up examinations up to 2 years when compared to baseline (*p* < 0.01).

**Table 1:** BDVA, height of SRF at the highest point and at the fovea, central retinal thickness as well as total retinal volume (mean ± sd) were measured at baseline and follow-up examinations up to 3 years

	Baseline (n = 60)	1 Month (n = 58)	p	3 Months (n = 55)	p	6 Months (n = 40)	p	1 Year (n = 31)	p	2 Years (n = 15)	p	3 Years (n = 10)	p
**BDVA (logMAR)**	0.24 ± 0.26	0.17 ± 0.25	0.014	0.13 ± 0.21	< 0.001	0.11 ± 0.23	0.002	0.05 ± 0.16	< 0.001	0.16 ± 0.26	0.421	0.09 ± 0.24	0.606
**Height of SRF at the highest point (µm)**	169 ± 103	82 ± 87	< 0.001	77 ± 75	< 0.001	87 ± 115	< 0.001	56 ± 87	< 0.001	51 ± 55	0.008	42 ± 64	0.005
**Height of SRF at the fovea (µm)**	139 ± 121	69 ± 98	< 0.001	52 ± 73	< 0.001	55 ± 101	< 0.001	30 ± 79	< 0.001	34 ± 44	0.011	33 ± 65	0.028
**cRT in central 1 mm (µm)**	378 ± 124	301 ± 99	< 0.001	291 ± 79	< 0.001	294 ± 107	0.001	264 ± 77	< 0.001	262 ± 57	0.006	269 ± 72	0.722
**Total retinal volume (mm**^**3**^)	9.40 ± 1.16	8.85 ± 0.92	< 0.001	8.85 ± 0.92	< 0.001	8.83 ± 0.77	0.001	8.65 ± 0.90	< 0.001	8.57 ± 0.46	0.001	8.70 ± 0.40	0.005

Wilcoxon rank test was used, *p* values are shown for matched samples compared to baseline

The distribution of eyes according to SRF response is presented in Table 2. At 1 month, 15 (26%) of eyes demonstrated complete fluid resolution and 24 (41%) partial fluid resolution from baseline. At one year 16 (52%) eyes achieved complete resolution, 7 (23%) had shown partial SRF resolution. At one year 24 (77%) of eyes were free of SRF at the fovea.

**Table 2:** Classification of eyes according to subretinal fluid (SRF) response and absence of foveal fluid over time

Visit	Classification according to SFR resolution at highest point			No fluid at the fovea
	Complete resolution	Partial resolution	Non-responder	
1 Month	15/58 (26%)	24/58 (41%)	19/58 (33%)	22/58 (38%)
3 Months	17/55 (31%)	17/55 (31%)	21/55 (38%)	28/55 (51%)
6 Months	13/40 (33%)	13/40 (33%)	14/40 (34%)	25/40 (63%)
1 Year	16/31 (52%)	7/31 (23%)	8/31 (25%)	24/31 (77%)
2 Years	6/15 (40%)	3/15 (20%)	6/15 (40%)	7 (47%)
3 Years	5/10 (50%)	4/10 (40%)	1/10 (10%)	7 (70%)

Complete resolution – zero fluid in the macula; Partial resolution - > 25% reduction in SFR height at the highest point from baseline; Non-responder - ≤25% reduction in SRF height at the highest point from baseline. Absence of foveal fluid refers to no detectable SRF at the foveal center

**Fig. 2:**
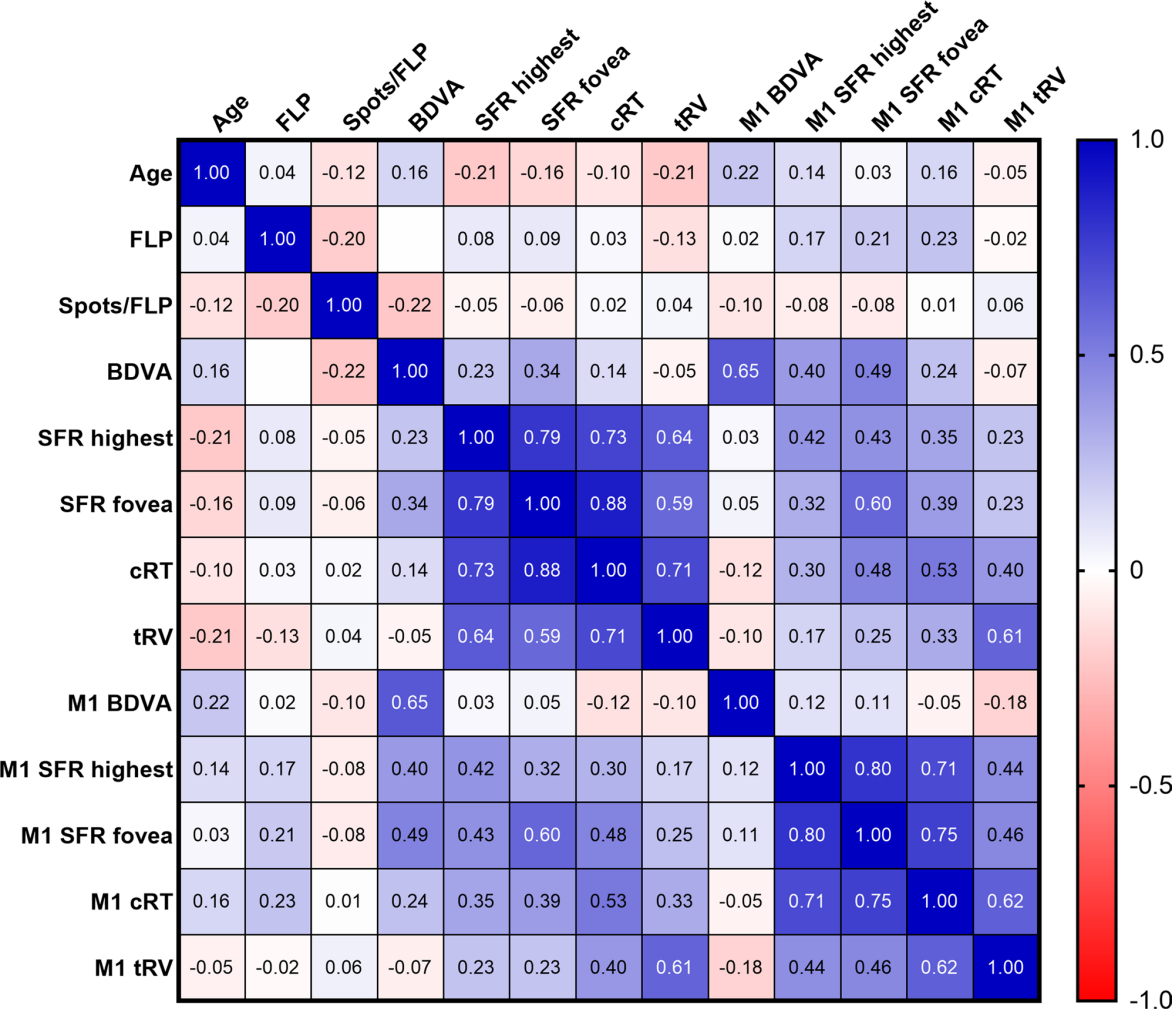
Correlation matrix between variables at baseline and the 1-month follow up. Spearman correlation coefficients (*r* values) are shown. Strong (r ≥ 0.6) and moderate (r ≥ 0.4) positive correlations are indicated by blue and light blue colors, respectively

Correlation analysis between various variables at baseline and the 1-month follow-up was performed, the results are shown in the correlation matrix (Fig. 2). Correlation analysis showed a moderate positive correlation between BDVA (logmar) and height of SRF at the fovea at baseline (*p* = 0.008). This shows that increased height of SRF at the fovea correlates to decreased visual acuity. BDVA showed no correlation with the height of SRF at the highest point, average RT in central 1 mm, and total retinal volume at baseline (*p* > 0.05).

The height of SRF at the highest point had a strong positive correlation with the height of SRF at the fovea (*p* < 0.001), as well as cRT (*p* < 0.001) and a moderate positive correlation with tRV (*p* < 0.001) at baseline and a moderate positive correlation with the height of SRF at the fovea (*p* = 0.001), as well as cRT (*p* = 0.006) at 1-month follow-up. There was no correlation between age, the amount of FLP or spots per FLP and other variables (*p* > 0.05). There was also no significant difference in BDVA, the height of SRF at the highest point, height of SRF at the fovea, cRT, tRV between gender at both the baseline and 1-month follow-up examinations (*p* > 0.05).

During the follow-up period, there were no adverse effects or complications, such as bleeding at the site of the test or treatment spots, observed.

### Re-treatment

Subjects were treated with SRT an average of 1.8 ± 1.0 times. 30 (50%) subjects were treated with SRT once, 19 (32%) twice, and 11 (18%) 3 times or more. During the follow-up period, 30 (50%) subjects required re-treatment. Re-treatment was performed on average 9.2 ± 9.6 months (minimum 1 and maximum 36 months) after the initial treatment.

One patient received re-treatment at the 1-month follow-up, 12 at the 3-month follow-up, 7 at the 6-month follow-up, 4 at the one-year follow-up, 4 at the 2-year follow-up, and 2 at the 3-year follow-up. At time of re-treatment 18 re-treated patients (60%) hat persistent and 12 (40%) had recurrent SRF. At the moment of re-treatment, 12 (40%) patients had persistent FLPs, 13 (43%) patients had recurring FLPs and 5 (17%) patients had a new FLPs. Two patients needed a second re-treatment at the 6-month follow-up, and three at the one-year follow-up. Out of these subjects, 1 had a persistent FLP, 2 recurring FLPs, and 2 new FLPs.

**Fig. 3:**
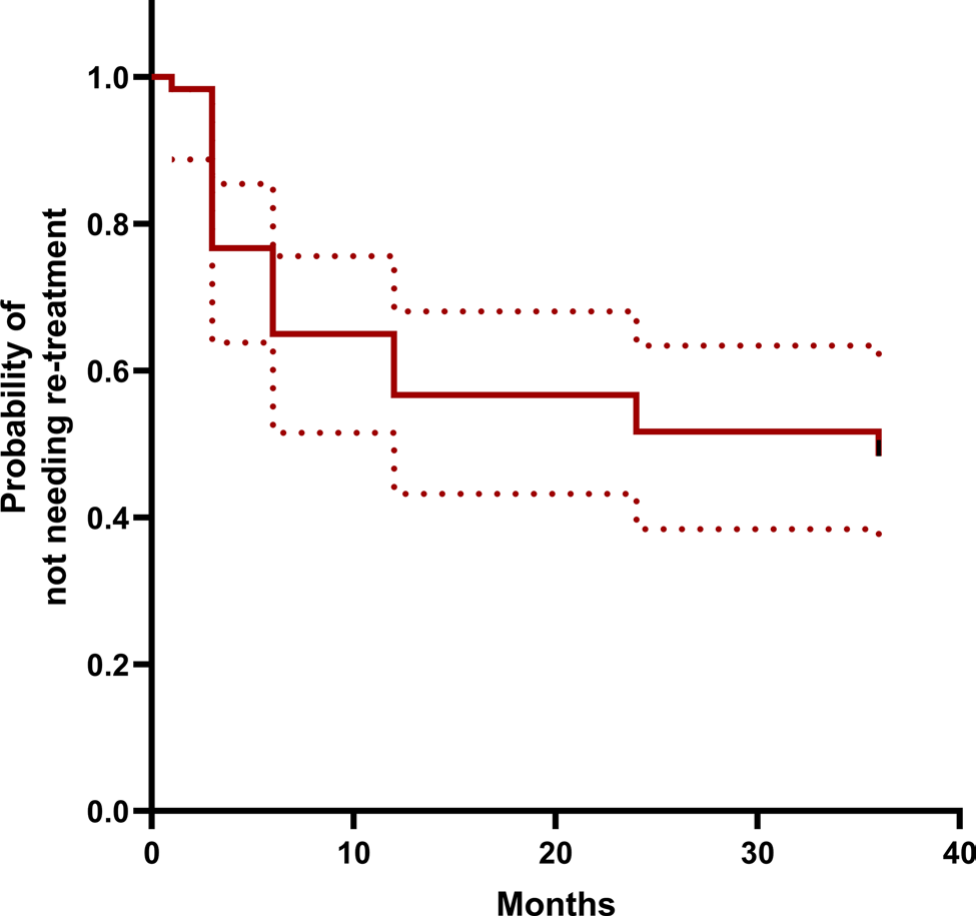
Kaplan-Meier curve showing the probability of not needing re-treatment over time. The dotted lines represent the 95% confidence interval

The Kaplan-Meier analysis revealed that the probability of not needing re-treatment was 77% at 3 months and 65% at 6 months (Fig. 3). By 12 months, the probability of re-treatment-free survival had decreased to 57%, by 2 years to 52%, and by 3 years to 49%. The results of Cox regression analysis showed that factors like age (hazard ratio 0.999; *p* = 0.951) and sex (hazard ratio 0.493; *p* = 0.706), BDVA at baseline (hazard ratio 2.182; *p* = 0.550), height of SRF at the fovea (hazard ratio 0.999; *p* = 0.655), amount of FLP treated (hazard ratio 0.783; *p* = 0.783) were not associated with the need for re-treatment.

We conducted a binary logistic regression analysis to evaluate predictors of re-treatment (Table 3). The results of the logistic regression analysis showed that age, sex, duration of symptoms, amount of FLPs or spots per FLP as well as distance from the fovea to the nearest FLP, BDVA, height of SRF at the highest point and fovea, cRT and tRV were not significant predictors for re-treatment (*p* > 0.05).

**Table 3:** Binary logistic regression analysis of predictors of re-treatment

	Odds ratio	95% Confidence interval		p value
		Lower	Upper	
**Age**	1.008	0.951	1.068	0.798
**Sex (male:female)**	0.246	0.033	1.849	0.173
**Duration of symptoms to SRT in months**	0.994	0.979	1.010	0.481
**Amount of FLPs**	0.786	0.299	2.067	0.626
**Spots/FLP**	1.170	0.968	1.413	0.104
**Distance from the fovea to the nearest FLP**	1.000	0.999	1.001	0.778
**BDVA**	0.332	0.21	5.220	0.433
**Height of SRF at the highest point**	1.004	0.990	1.018	0.604
**Height of SRF at the fovea**	1.007	0.986	1.030	0.502
**Central retinal thickness**	0.702	0.241	2.049	0.517
**Total retinal volume**	0.995	0.977	1.013	0.560

### Comparison between subjects receiving a single treatment and re-treatment

Symptom duration before initial SRT and subjective visual acuity differences between single and re-treated patients at follow-ups are shown in Table 4. Symptom duration before SRT did not differ significantly between single and re-treated groups (*p* = 0.410). Patients in the re-treatment group reported a subjective improvement of visual acuity at the 1-month follow-up examination more frequently than subjects in the single treatment group (*p* < 0.05). However, at the 3-month follow-up significantly more patients that needed re-treatment reported worsened vision (*p* < 0.05).

**Table 4:** Distribution of subjects by duration of symptoms to initial SRT and subjective difference between single treatment vs. re-treatment groups

	**Single treatment group**				**Re-treatment group**				
**Duration from beginning of symptoms to initial treatment with SRT**									
	** < 6 months**	**6–12 months**	**1–2 years**	** > 2 years**	** < 6 months**	**6–12 months**	**1–2 years**	** > 2 years**	**p**
Subjects, n (%)	3 (10%)	6 (20%)	7 (23%)	14 (47%)	7 (23%)	8 (27%)	5 (17%)	10 (33%)	0.410

**Subjective difference in visual acuity**									
	**Better**	**Same**	**Worse**	**No data**	**Better**	**Same**	**Worse**	**No data**	**p**
1-month	8 (28%)*	19 (66%)*	1 (3%)	1 (3%)	16 (54%)*	9 (30%)*	1 (3%)	4 (13%)	**0.028**
3-months	11 (44%)	11 (44%)	0 (0%)*	3 (12%)	10 (33%)	10 (33%)	9 (30%)*	1 (3%)	**0.010**
6-months	0 (0%)*	10 (77%)*	2 (15%)	1 (8%)	7 (26%)*	10 (37%)*	9 (33%)	1 (4%)	**0.039**
1-year	0 (0%)	6 (86%)	1 (14%)	0 (0%)	4 (17%)	11 (46%)	6 (25%)	3 (12%)	0.487
2-years	0 (0%)	2 (100%)	0 (0%)	0 (0%)	3 (22%)	5 (39%)	5 (39%)	0 (0%)	N.A.
3-years	0 (0%)	1 (100%)	0 (0%)	0 (0%)	0 (0%)	6 (67%)	1 (11%)	2 (22%)	N.A.

Fisher’s exact test was used test was used

*p < 0.05 Z-test used to compare proportions of variables between the single treatment and re-treatment groups

**Fig. 4:**
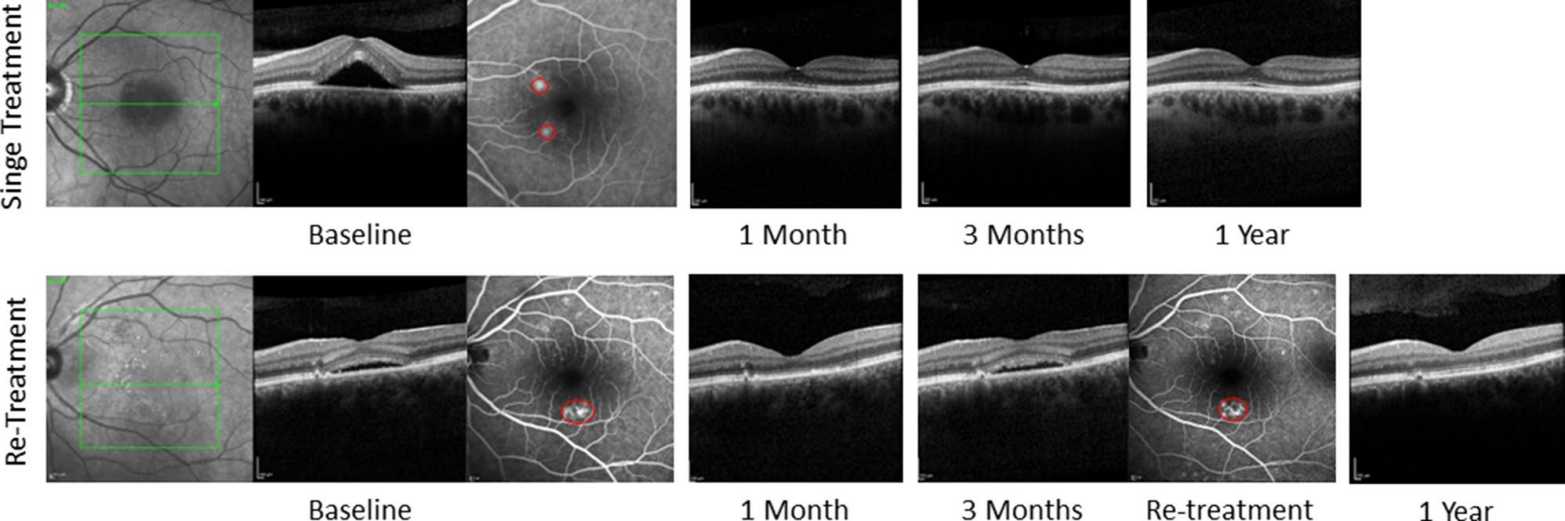
Multimodal imaging of two representative cases: single treatment versus re-treatment over one year.At baseline, both cases exhibited subretinal fluid in OCT and a focal leakage point in FA; therefore, selective retina therapy was performed. Treatment areas are indicated by red circles on fluorescein angiography. In the single-treatment case, SRT at baseline resulted in complete fluid resolution at 1 month, which persisted throughout follow-up. In the re-treatment case, recurrent subretinal fluid and a reappearing focal leakage point were observed at 3 months, prompting re-treatment. Imaging at 1 year demonstrated complete resolution of subretinal fluid in both cases

BDVA, SRF height (peak and foveal), cRT, and tRV were measured at baseline and follow-ups for single and re-treated groups (Table 5). Significant improvement in BDVA in the single treatment group was only observed at the 3-month follow-up examination compared to baseline (*p* = 0.038) whereas BDVA improved in every follow-up examination up to 1-year in the re-treatment group (*p* < 0.05) but showed no difference at the 2-year and 3-year examinations when compared to baseline. Due to a small sample size of patients from the single treatment group, significance in these values between baseline and 2-year and 3-year follow-up was not analyzed. Figure 4 shows representative single and re-treatment cases. At 3 months, SRF height (peak and at the fovea) was significantly higher in the re-treatment group than in the single-treatment group (*p* = 0.011).

**Table 5:** BDVA, height of SRF at the highest point and at the fovea, central retinal thickness as well as total retinal volume (mean ± sd) were measured at baseline and follow-up examinations up to 3 years in subjects that received a single treatment and re-treatment

Single treatment group													
	Baseline (n = 30)	1 Month (n = 29)	p	3 Months (n = 25)	p	6 Months (n = 13)	p	1 Year (n = 7)	p	2 Years (n = 2)	p	3 Years (n = 1)	p
**BDVA (logMAR)**	0.24 ± 0.31	0.21 ± 0.30	0.267	0.13 ± 0.25	0.038	0.08 ± 0.17	0.089	0.04 ± 0.09	0.172	0.01 ± 0.13	N.A.	0.15	N.A.
**Height of SRF at the highest point (µm)**	109 ± 96	65 ± 68	< 0.001	49 ± 58*	< 0.001	77 ± 93	0.019	32 ± 46	0.034	0 ± 0	N.A.	0	N.A.
**Height of SRF at the fovea (µm)**	131 ± 126	57 ± 96	< 0.001	34 ± 75*	< 0.001	54 ± 92	0.038	16 ± 41	0.043	0 ± 0	N.A.	0	N.A.
**Average RT in central 1 mm (µm)**	370 ± 129	294 ± 95	< 0.001	277 ± 79	< 0.001	302 ± 99	0.028	258 ± 59	0.128	240 ± 15	N.A.	232	N.A.
**Total retinal volume (mm**^**3**^)	9.35 ± 1.16	8.74 ± 0.77	< 0.001	8.75 ± 0.45	< 0.001	8.69 ± 0.72	0.021	8.26 ± 0.60	0.028	8.32 ± 0.18	N.A.	8.18	N.A.

**Re-treatment group**													
	**Baseline** **(n = 30)**	**1 Month** **(n = 30)**	p	**3 Months** **(n = 30)**	p	**6 Months** **(n = 27)**	p	**1 Year** **(n = 24)**	p	**2 Years** **(n = 13)**	p	**3 Years** **(n = 9)**	p
**BDVA (logMAR)**	0.22 ± 0.22	0.14 ± 0.18	0.019	0.13 ± 0.19	0.006	0.13 ± 0.25	0.014	0.05 ± 0.18	0.002	0.18 ± 0.27	0.789	0.08 ± 0.25	0.590
**Height of SRF at the highest point (µm)**	178 ± 111	100 ± 101	< 0.001	101 ± 79*	0.002	92 ± 125	0.004	63 ± 96	0.001	59 ± 55	0.025	47 ± 66	0.008
**Height of SRF at the fovea (µm)**	147 ± 117	82 ± 99	< 0.001	66 ± 69*	0.001	56 ± 107	0.005	34 ± 87	0.001	39 ± 45	0.033	37 ± 68	0.050
**Average RT in central 1 mm (µm)**	386 ± 120	308 ± 104	< 0.001	302 ± 79	0.002	291 ± 112	0.007	266 ± 82	0.001	265 ± 61	0.019	273 ± 75	0.015
**Total retinal volume (mm**^**3**^)	9.44 ± 1.18	8.96 ± 1.05	< 0.001	8.77 ± 0.67	0.001	8.90 ± 0.79	0.012	8.77 ± 0.95	0.001	8.61 ± 0.48	0.002	8.75 ± 0.38	0.008

Wilcoxon rank test was used, *p* values are shown for matched samples compared to baseline

*p = 0.011 – Mann-Whitney U test comparing single treatment vs. re-treatment groups

N.A. – not applicable

Subjects that received a single SRT treatment received an average of 7.3 ± 3.0 spots per FLP and subjects that needed re-treatment received an average of 9.0 ± 4.5 spots per FLP (*p* = 0.70). In the re-treatment group, subjects that had a persistent FLP (*n* = 12) received an average of 9.2 ± 5.0 irradiation spots per FLP, and the ones with a recurrent FLP (*n* = 13) had a mean of 8.6 ± 4.6 irradiation spots per FLP and subjects with a new FLP (*n* = 5) had a mean of 9.7 ± 3.8 irradiation spots per FLP performed (*p* = 0.889). There was no significant difference in age when comparing between patients that were treated a single time and re-treated patients (48.8 ± 9.7 vs. 47.7 ± 12.7 respectively, *p* = 0.724).

## Discussion

The goal of this study was to examine the clinical impact of SRT in patients with cCSCR, including the necessity for re-treatment and the factors associated with it. The study found that height of SRF, cRT, and tRV decreased over time, accompanied by a significant improvement in BDVA within the first year of follow-up. It is important to highlight that over the three-year follow-up period, half of the participants required re-treatment, mostly because of chronic or recurrent FLPs.

The BDVA in logMAR improved from 0.24 (Snellen equivalent: 20/34.8) at the start of the study to 0.05 (Snellen equivalent: 20/22.4) after one year in our cohort of subjects with cCSCR. At the 2-year and 3-year follow-ups, a significant decrease in SRF both at the highest point and at the fovea as well as reduced total retinal volume were observed. However, the BDVA showed no significant difference when compared to the baseline due to good initial visual acuity and high variance and large confidence intervals in these small patient groups.

This study lacked a control group, but comparisons can be made with existing data on untreated CSCR. The VICI trial reported an increase in visual acuity from 0.14 (Snellen equivalent: 20/27.6) to 0.11 (Snellen equivalent: 20/25.8) after 12 months in the control group and from 0.16 (Snellen equivalent: 20/28.9) to 0.09 (Snellen equivalent: 20/24.6) in the treatment group [6]. The study found no significant difference in the increase of visual acuity between the eplerenone therapy and placebo groups. Therefore, the visual acuity improvement of both of these groups can be used as a benchmark for therapy-naive CSCR. Another smaller study observed a spontaneous increase in visual acuity from 0.28 (Snellen equivalent: 20/38.1) to 0.14 (Snellen equivalent: 20/27.6) in 5 eyes with CSCR over a one-year period [23]. When comparing our results to the findings of these studies reporting on the natural course of CSCR, we observed a greater short-term improvement in BDVA after SRT. However, given the retrospective design, high loss to follow-up, and potential selection bias, these functional outcomes must be interpreted with caution. Prospective randomized studies are needed to confirm the efficacy of SRT on visual function. Furthermore, a network meta-analysis indirectly compared SRT to placebo for the treatment of CSCR and revealed a significant effect on SRF resolution [24]. However, a comparison to established PDT would have been of particular interest.

The PLACE study, a large randomized controlled study on CSCR, reported an increase in visual acuity both in the micropulse laser group and in the PDT group after 8 months [5]. Unfortunately, the 8-month study period falls between the 6 and 12-month follow-up periods examined in this study, which makes it difficult to compare these two investigations. Nevertheless, SRT appears to demonstrate comparable anatomic results to micropulse laser therapy. However, PDT is a standardized, guideline-based treatment with well-established dosing protocols, whereas many aspects of micropulse laser therapy and SRT application remain to be defined. In SRT, spot pattern, density, and retreatment criteria are not yet standardized and likely influenced outcomes in our study. The lack of studies on the course of untreated CSCR shows the need for more research in this area. This highlights the importance and potential of unified patient registries and we look forward to the first results of the recently created CSCR registry [25].

PDT with verteporfin is the most established therapy for CSCR at this time. However, the supply of verteporfin is occasionally limited, which can delay treatment in some cases [26]. In addition, although infrequently, PDT can cause adverse effects. These include nausea, headache, dyspnoea, syncope, dizziness, and temporary renal artery stenosis [2]. In longer observation periods RPE and choroidal ischemia were observed after treatment with PDT [13]. Furthermore, pain, edema, inflammation, extravasation and hypersensitivity reactions can be triggered by the injection of verteporfin [2]. PDT is contraindicated in cases of pregnancy, porphyria, and impaired liver function. [2]. In comparison, SRT is a promising treatment option, because it has no contraindications as well as no side effects that have been reported in previous studies to date or observed in this study. Most importantly, no atrophies in the RPE and neuroretina could be detected following SRT, even over an extended three-year follow-up period [18]. Only overtreatment during SRT may result in chorioretinal scarring, such as in photocoagulation [14].

The energy-level applied should be just as high as it is needed to selectively rupture RPE cells, then followed by regeneration. This can be securely achieved by some kind of dosimetry. Titration, as applied here, is common. Dosimetry feedback, like optoacoustic feedback is desired and implemented in the used system [14, 27]. However, the most effective application pattern, spot density and spacing of the SRT laser spots at the leakage point as well as the number of treatments needed for the treatment of CSCR and other macular diseases are unknown. Alterations of the treatment pattern might improve results.

Some groups always use a minimum spacing of one laser spot diameter between laser spots during SRT, while others use less than one diameter [7, 13, 28–30]. Additionally, some researchers suggest using a predetermined minimum number of laser foci per FLP [7]. To our knowledge, only one study has examined the effect of laser spot spacing on the retina, showing that smaller distances result in larger lesions [31]. In both low-density (LD) and high-density (HD) SRT patterns, including no-spacing pattern, RPE defects were closed by new polygonal RPE cells with microvilli, though closure took longer after HD patterns. Compared to LD-SRT, HD-SRT appears more effective at inducing broader RPE rejuvenation, greater protein expression changes, and restoring larger areas of normal-appearing RPE with microvilli, without gaps between treated zones [15]. Therefore, high-density or no-spacing patterns are recommended [31].

We applied a pattern of a single SRT spot at the center of the RPE leakage area, with additional laser spots arranged in a circular pattern with spacing ranging from half a spot diameter to confluent. This approach aligns with treatment patterns described in the literature [32, 33]. Using this pattern, no correlation between the applied laser spots per FLP and any other variable could be found.

As for the number of treatments needed, our re-treatment rate was 50%, with most of these subjects requiring re-treatment after three months. This could indicate undertreatment, as 40% of subjects that needed re-treatment had persistent FLPs, 43% recurrent FLPs, and only 5 cases new FLPs (17%).

Our study found that re-treated patients had significantly higher SRF height at the 3-month follow-up compared to those who received only a single SRT session. This highlights the critical role of OCT monitoring at 3-months to ensure timely re-treatment. Many studies describe varying re-treatment algorithms, some showing promising results. Some involve treating any FA-identified leakage, while others follow fixed intervals, such as 6–12 weeks before re-treatment [7, 13, 21, 34]. Moreover, our cohort was likely undertreated. In some cases, re-treatment was withheld when residual fluid was present but no visible focal leakage point could be identified, and a conservative re-treatment policy was applied overall. These factors may have limited the efficacy observed in this study.

Given the variability of therapy regimens, it is essential to establish a consensus. As with intravitreal injection in other indications, a treatment scheme such as the pro re-nata (PRN) approach is advisable [35]. Since irreversible damage to the photoreceptor layer can occur in CSCR due to persistent fluid after three months, a zero-fluid tolerance is a reasonable re-treatment criterion [3]. The high rate of necessary re-treatments in our study may be attributed to suboptimal treatment caused by the aforementioned spot pattern. Furthermore, it is plausible that the high recurrence rate is linked to the chronic nature of CSCR and the fact that SRT solely addresses the affected retinal RPE, leaving choroidal impairment untreated [2, 19]. This may also support a PRN-based SRT strategy.

Other studies show that patients with smoking history and diffuse leakage type on FA may be at greater risk of persistent SRF [29]. Therefore, they could be more likely to require re-treatment. Another study identified baseline SRF height as a significant predictor for re-treatment after SRT in CSCR [34]. Additionally, factors such as best corrected visual acuity, age, gender, symptom duration, previous history of intravitreal bevacizumab injection, type of leakage, and presence of PED were not predictive [34]. Our analysis revealed that none of the baseline factors we examined significantly predicted re-treatment need. However, we did not account for risk factors, such as smoking.

The present study has two major limitations: the lack of a control group and a small sample size at 2- and 3-year follow-ups, due to these being voluntary examinations. The absence of a placebo control has been previously elaborated in the discussion. Regarding the small sample at later follow-ups, this, along with the wide confidence interval, may explain the non-significant BDVA increase. While this complicates statistical analysis, mean values suggest good visual acuity at these time points. Moreover, significant reductions in SRF height (peak and foveal) and total retinal volume were observed, accompanied by functional improvement in BDVA within the first year. While these findings suggest a therapeutic benefit of SRT, SRF reduction alone cannot be equated with treatment success, as spontaneous improvement is also known in cCSCR. Given the retrospective nature of our study and lack of a control group, our results should be interpreted carefully and warrant confirmation in prospective randomized trials.

A notable loss to follow-up occurred in the single-treatment group, which showed significant BDVA and SRF improvement at 3 months. This attrition may partly explain the lack of significant BDVA changes in that group at 6 months and beyond. Additionally, patients reporting subjective visual improvement may have been less likely to attend follow-up visits, which likely contributed to attrition already within the first year and further reduced case numbers at 2- and 3-year visits. Given the long travel times and the well-established structure of outpatient ophthalmology care, particularly in rural regions, those patients might have preferred to continue follow-up locally. This selection bias complicates the interpretation of long-term outcomes.

Another limitation was not adopting a zero-fluid policy, opting instead for a more reserved approach. Due to the retrospective nature of the study and the cautious re-treatment policy at that time as well as due to the chosen SRT pattern and spot density, which might have been suboptimal, we believe that some patients were undertreated, which may have reduced the potential functional and anatomical benefits of SRT in our cohort. Given the promising re-treatment outcomes in this and other studies [21], earlier re-treatment in cases of residual SRF might have improved long-term results.

In addition, there is inherent variability in manual spacing during slit-lamp–based SRT, caused by small eye movements and limited intraoperative lesion visibility. While overlap was consistently avoided, spacing accuracy remains a known limitation of manual SRT application.

Another limitation is that potential confounders such as smoking status, corticosteroid exposure, and systemic comorbidities were not systematically recorded in this retrospective dataset. These factors may have influenced both the natural course of disease and treatment outcomes and should be controlled for in future prospective studies.

The next important step for establishing SRT is to explore the optimal pattern, spot number, and spacing at the FLP needed to treat CSCR, as well as the required number of treatments. Thus, we hope for a future phase 2 randomized trial comparing high- vs. low-density SRT in CSCR patients, both following a PRN zero-fluid-tolerance scheme with 3-month control visits. With the resulting dosing data, a large randomized controlled trial could compare SRT and PDT, providing final insight into the efficacy of both treatments.

## Conclusions

SRT appears to be a safe treatment option for cCSCR. Our findings show anatomical and functional benefits and re-treatment has proven to be beneficial in insufficiently treated patients or recurring cases. Due to the retrospective design, high dropout rate, and conservative re-treatment approach, these results should be interpreted with caution. Although there is still debate on when re-treatment should be performed, we recommended to perform regular check-ups every three months and perform re-treatment in cases of persistent or recurring SRF following a zero-fluid tolerance policy.

## Data Availability

The datasets used and/or analysed during the current study are available from the corresponding author on reasonable request.

## Abbreviation

BDVA: Best Documented Visual Acuity
cCSCR: Chronic Central Serous Chorioretinopathy
cRT: Central Retinal Thickness
CSCR: Central Serous Chorioretinopathy
ETDRS: Early Treatment Diabetic Retinopathy Study
FA: Fluorescein Angiography
FAF: Fundus Autofluorescence
FLP: Focal Leakage Point
HD-SRT: High-Density Selective Retina Therapy
LD-SRT: Low-Density Selective Retina Therapy
logMAR: Logarithm of the Minimum Angle of Resolution
Nd:YLF: Neodymium-doped Yttrium Lithium Fluoride
OCT: Optical Coherence Tomography
PDT: Photodynamic Therapy
PRN: Pro re Nata (as needed)
PTL: Photothermal Laser Therapy
RELITE: Regenerative Retinal Laser and Light Therapies
RPE: Retinal Pigment Epithelium
SD-OCT: Spectral-Domain Optical Coherence Tomography
SRT: Selective Retina Therapy
SRF: Subretinal Fluid
SPSS: Statistical Package for the Social Sciences
tRV: Total Retinal Volume
